# Propagation behavior of coal crack induced by liquid CO_2_ phase change blasting considering blasting pressure effects

**DOI:** 10.1371/journal.pone.0313360

**Published:** 2024-11-15

**Authors:** Shu Ma, Guiming Li, Yongjiang Zhang, Hao Liu

**Affiliations:** 1 School of Safety Engineering, China University of Mining and Technology, Xuzhou, China; 2 Guizhou Energy Industry Research Institute Co., Ltd., Guiyang, China; 3 School of Mechanics and Civil Engineering, China University of Mining and Technology (Beijing), Beijing, China; 4 China Coal Technology and Engineering Group Chongqing Research Institute, Chongqing, China; 5 College of Aerospace Engineering, Chongqing University, Chongqing, China; 6 Chongqing Key Laboratory of Heterogeneous Material Mechanics, Chongqing University, Chongqing, China; Shenyang Jianzhu University, CHINA

## Abstract

To investigate the crack propagation mechanisms in low-permeability coal seams induced by liquid CO_2_ phase change blasting under different blasting pressures, this research presents an experimental study conducted on a small liquid CO_2_ phase change blasting test system. The failure mode, crack morphology, and distribution characteristics of the coal rock model specimens under different liquid CO_2_ phase change blasting pressure were revealed, analyzing the crack shapes and expansion process. The results show that with increasing blasting pressure, both the number and complexity of cracks significantly increase under liquid CO_2_ phase change blasting, evolving from simple linear cracks to more complex multi-directional networks. Furthermore, the process of crack generation and expansion during liquid CO_2_ phase change blasting in coal and rock is controlled by the interaction of shock waves and quasi-static stress resulting from high-pressure CO_2_ phase transition in the borehole. Cracks form in distinct zones: the broken zone, where shock waves cause severe crushing near the borehole; the crack zone, where quasi-static tensile stress drives crack propagation. Higher confining and CO_2_ blasting pressures increase crack propagation. The research results offer valuable insights for optimizing blasting design in liquid CO_2_ phase change fracturing.

## 1 Introduction

Coal mines in China primarily utilize underground mining. As the mining depth increases at a rate of 10–20 meters per year, the geological conditions of coal seams become more complex [1–5]. Specifically, the gas pressure and gas content of deep coal seams are on the rise, while the permeability of coal seams is gradually decreasing [6–10]. Consequently, gas outbursts or gas emission accidents are likely to occur during coal mining [11–14]. Gas accidents have long been the most detrimental in coal mine production accidents, and the prevention and control of gas disasters remain the primary task for China’s coal safety production [15–18].

Gas extraction is the most direct and effective measure to prevent gas disasters and avoid air pollution [19–22], and it is also the key technology for realizing the high-value utilization of gas [23–26]. Enhancing the permeability of the coal seam is crucial for improving the gas extraction rate and preventing gas disasters [27,28]. Currently, the methods to increase the permeability of coal seams mainly include pre-splitting blasting [29], hydraulic slotting [30], hydraulic fracturing [31], hydraulic punching [32], among others [33–35]. The essence of these methods is to improve the crack system of coal seams locally through physical and chemical manual intervention, thereby improving the permeability of coal seams and enhancing the effect of gas drainage [36]. However, traditional deep-hole pre-splitting blasting, which uses coal mine explosives as power, has issues such as explosion risk, complex operation, and explosive control [37,38]. Hydraulic anti-reflection technology, including hydraulic slotting, hydraulic fracturing, and hydraulic punching, is currently the most important technical method to strengthen gas extraction [39,40]. However, it has some problems such as difficult operation, water accumulation in roadways, poor working environment, and chemical reagents can cause pollution to groundwater [41–43]. These drawbacks greatly limit the popularization and application of these technologies [44]. Liquid CO_2_ phase change blasting fracturing technology was originally used in foreign countries for boiler cleaning, building demolition, special area blasting among other fields. Due to its advantages of adjustable and controllable fracturing energy, low environmental pollution, high safety, and no need to consume water resources [45,46], it has also received attention in the aspect of anti-reflection of low-permeability coal seams.

Liquid CO_2_ phase change blasting fracturing technology originated in the United States, Britain, France, and other industrially developed Western countries in the 1960s. Due to the intrinsic safety characteristics of this physical phase change blasting fracturing process, it is used for directional rock cutting in quarries, descaling in cement plants, and improving ore particle size [47,48]. In 1983, using CO_2_ fracturing instead of traditional hydraulic technology to increase the permeability of coal seams was first proposed. In the mid-1990s, China began to introduce CO_2_ phase change blasting fracturing technology. This technology was first used to increase the permeability of low-permeability coal seams in 2015 [49,50]. Through experimental research in Jiulishan Coal Mine and Pingmei No.13 Coal Mine of Henan Coking Coal Energy Co., Ltd., it was found that CO_2_ phase change blasting fracturing has a very significant effect on the permeability of coal seams. Subsequently, many coal mines in China have carried out CO_2_ anti-reflection fracturing coal seam experiments [51–53], which confirmed the feasibility of this technology to increase the permeability of coal seams. Therefore, existing research indicates that CO_2_ phase change blasting fracturing technology is less constrained by the geological conditions of the coal seam, and possesses characteristics such as safety, portability, controllability, high efficiency, low cost, and wide applicability [54,55]. It holds significant potential for application in the low permeability and high gas coal seams of deep coal mines in China. However, the field experiments also highlighted certain limitations, such as challenges in optimizing blasting parameters under varying geological conditions, and difficulties in achieving consistent fracturing efficiency in deeply buried and highly gas-rich coal seams. This suggests the need for further investigation into refining the application process. Consequently, this study seeks to address these limitations through laboratory experiments with different blasting pressure, aiming to optimize the fracturing parameters and evaluate the performance of CO_2_ phase change blasting in complex geological settings.

Moreover, understanding the crack propagation law of coal in the process of CO_2_ phase change blasting and permeability enhancement is of positive significance for improving the effect of CO_2_ phase change blasting [56–58]. Jia et al. [59,60] used ANSYS / LS-DYNA, RFPA2D-Flow, FLAC3D, and other software to simulate the evolution law of coal and rock cracks around the borehole, and determined the optimal layout parameters of blasting holes. The permeability of the coal seam in an industrial test at Mabao Coal Mine has been greatly improved. Mi et al. [61] studied the influence of liquid CO_2_ injection pressure, fracturing fluid viscosity, and crack occurrence on crack propagation, and found that the crack propagation radius was positively correlated with injection pressure and crack occurrence, and negatively correlated with fracturing fluid viscosity. Wang et al. [62] investigated the radial vibration characteristics of a coal briquette under the action of a CO_2_ gas explosion, with a focus on analyzing the vibration signals and energy distribution throughout the explosion’s life cycle. A radial vibration mechanical model was constructed, and laboratory tests were conducted to quantify the vibration parameters, such as acceleration and velocity, during three distinct stages of the CO_2_ gas explosion: the stress wave action stage, the energy storage stage, and the gas splitting stage. Through physical experiments, Zhou et al. [63] investigated the law of supercritical CO_2_ fracturing on specimen fracture under temperature effect. According to the temperature effect, the crack patterns were categorized into inclined cracks, planar cracks and crack networks. In summary, while these studies have advanced our understanding of CO_2_ phase change blasting, particularly in field applications and some laboratory-scale tests [64], research into the detailed distribution and extension of cracks remains limited. Notably, studies exploring the impact of varying blasting pressures are scarce. This gap highlights the need for further investigation into how different blasting pressures influence crack propagation, a focus that this study aims to address through controlled experiments.

Although significant progress has been made in understanding liquid CO_2_ phase change blasting fracturing technology through field applications, the detailed mechanisms of crack initiation and propagation remain insufficiently explored. While existing studies offer a general understanding of the effects of CO_2_ phase change blasting, there is a lack of in-depth research into how varying blasting pressures specifically influence crack development and expansion. This gap in knowledge is critical, as optimizing blasting pressure is key to enhancing the efficiency and effectiveness of the technology. Consequently, further investigation into the impact of different blasting pressures on crack propagation is essential for improving CO_2_ phase change blasting performance. Therefore, by studying the distribution law of surface cracks after liquid CO_2_ phase change blasting fracturing, the mechanism of crack generation and expansion is further elucidated, providing a scientific basis for optimizing blasting design in liquid CO_2_ phase change fracturing.

## 2 Materials and methods

### 2.1 Overview of the mine

The samples prepared in this experiment were based on the #9 coal seam of Guizhou Linhua Coal Mine. Linhua Coal Mine is located in the southwest of Jinsha County, Guizhou Province, with a mining area of 21.85 km^2^. The coal-bearing strata belong to the Longtan Formation, with a moderate degree of geological structure and five coal seams available for extraction. Among them, the burial depth of the #9 coal seam ranges from 393 to 511 meters, with an average thickness of 3.01 meters and a coal seam dip angle of 11°. The distances between the upper and lower coal seams are 14 meters and 32 meters, respectively. The immediate roof of this coal seam consists of sandy mudstone, while the immediate floor is composed of mudstone and sandy mudstone. Additionally, the gas content of the coal seam ranges from 20.82~23.42 m^3^/t, with gas pressure ranging from 0.63~2.15 MPa, indicating a tendency for coal and gas outbursts. Furthermore, while the #9 coal seam is rich in gas, its permeability coefficient ranges from 0.05~7.17 m^2^/(MPa^2^·d).

Currently, the main challenges facing gas extraction from the #9 coal seam at Linhua Coal Mine include: ① low permeability of the coal seam, leading to a large volume of gas extraction engineering; ② high engineering costs with unsatisfactory extraction results; ③ low gas extraction concentration, resulting in inefficient utilization. Therefore, it is necessary to conduct experiments on liquid CO_2_ phase change blasting fracturing and permeability enhancement under the aforementioned background to improve the efficiency of gas extraction from the coal seam.

### 2.2 Preparation of samples

To minimize the anisotropy of coal and the interference of primary cracks on the experimental results, this experiment plans to use model specimens to conduct crack initiation and expansion experiments of liquid CO_2_ phase change blasting coal under triaxial stress conditions.

**Fig 1:**
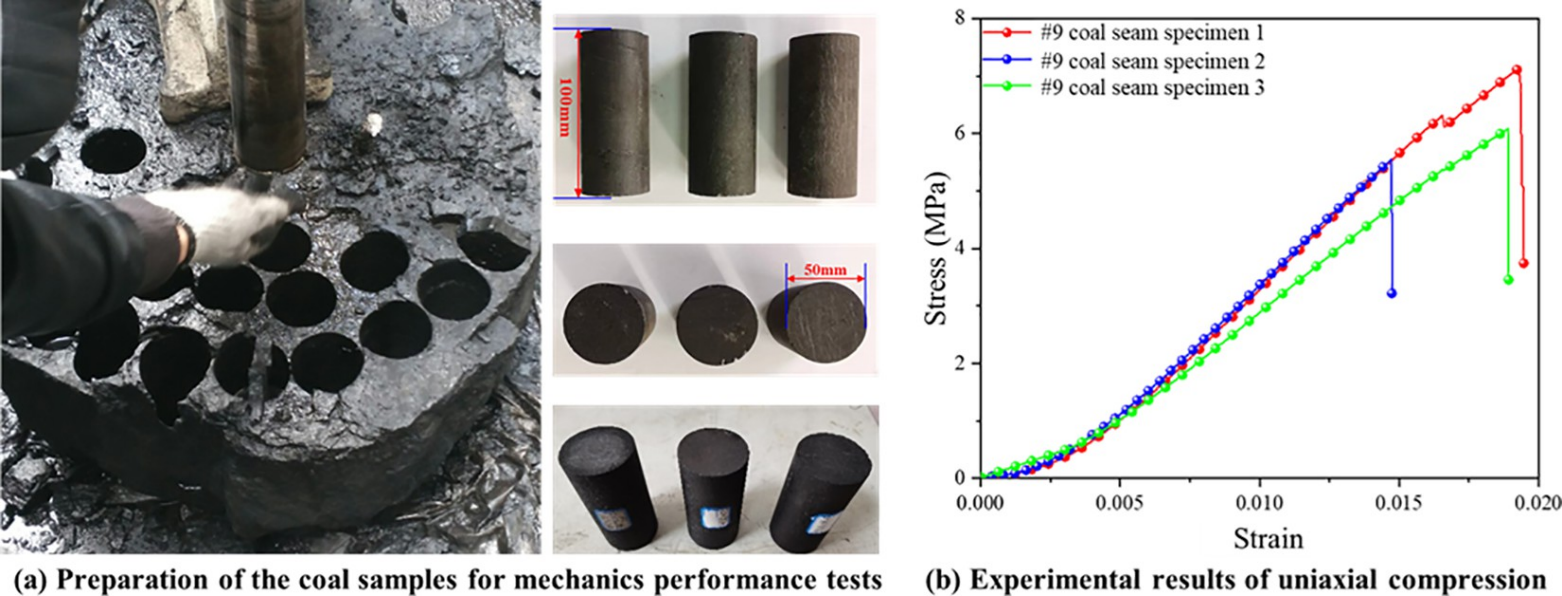
Mechanical properties testing of raw coal seam. (a) Preparation of the coal samples for mechanics performance tests; (b) Experimental results of uniaxial compression.

Before the experiment, the #9 coal seam of Linhua Mine was cut and polished to make a standard specimen with a diameter of 50mm and a height of 100mm, as shown in Fig 1A. Due to the various anisotropy of the coal specimens, in order to avoid different test results caused by individual differences of the specimens, the coal samples of the coal seams were taken into three samples for the test of the compressive strength, and the coal specimens for each test were taken from the same coal block. The results of the three uniaxial compressive strength tests are shown in Fig 1B. At the initial loading, the primary fractures inside the specimen closed, and the specimen was gradually compacted (compaction stage); followed by the stable development stage of the fractures, then microcracks were formed inside the specimen, and the local load-bearing capacity decreased, but the overall growth capacity was still on the rise (microcrack expansion stage); when the stress reached the peak, the stress dropped sharply, and the specimen was destroyed (instability destruction stage). The analysis shows that the strains of the three coal specimens in the #9 coal seam are 0.014, 0.018 and 0.019, respectively, and the corresponding peak stresses are 5.58, 6.87 and 5.84 MPa, respectively, and the average value of the three specimens’ compressive strengths is 6.10 MPa.

Due to the high cost of collecting large coal specimens in the field and the complexity of the transportation and preservation process, for this reason, this experiment uses the method of similar material simulation to conduct the experiment. This method not only reduces the cost and operation difficulty, but also reflects the experimental results more stably and accurately, and improves the controllability and reliability of the experiment. The model specimens used cement, gypsum, and sand as the basic materials, with a cementing agent or cement as auxiliary materials [65]. As the experimental study focuses on the crack initiation and expansion of similar materials, it is crucial to ensure that the compressive strength, density, and other properties of the model specimen match those of the coal rock specimen [66,67]. To obtain physical and mechanical properties similar to the #9 coal seam of Linhua Coal Mine, the material ratio used in this experiment was determined to be sand:cement:gypsum = 1:1:1.5 [68–72]. By conducting mechanical properties and weighing tests on model samples and raw coal samples of the same volume under this ratio, it was found that the error between the two can meet the accuracy requirements in engineering. The mechanical properties of the fabricated model specimens are shown in Table 1. The strength of the model specimens is consistent with the coal specimens taken in the field.

**Table 1:** Mechanical performance parameters of material.

Parameter	Compressive strength (MPa)	Elastic modulus (MPa)	Tensile strength (MPa)	Cohesion (MPa)	Angle of internal friction (°)
Value	6.34	1229	1.67	2.01	28.03

**Fig 2:**
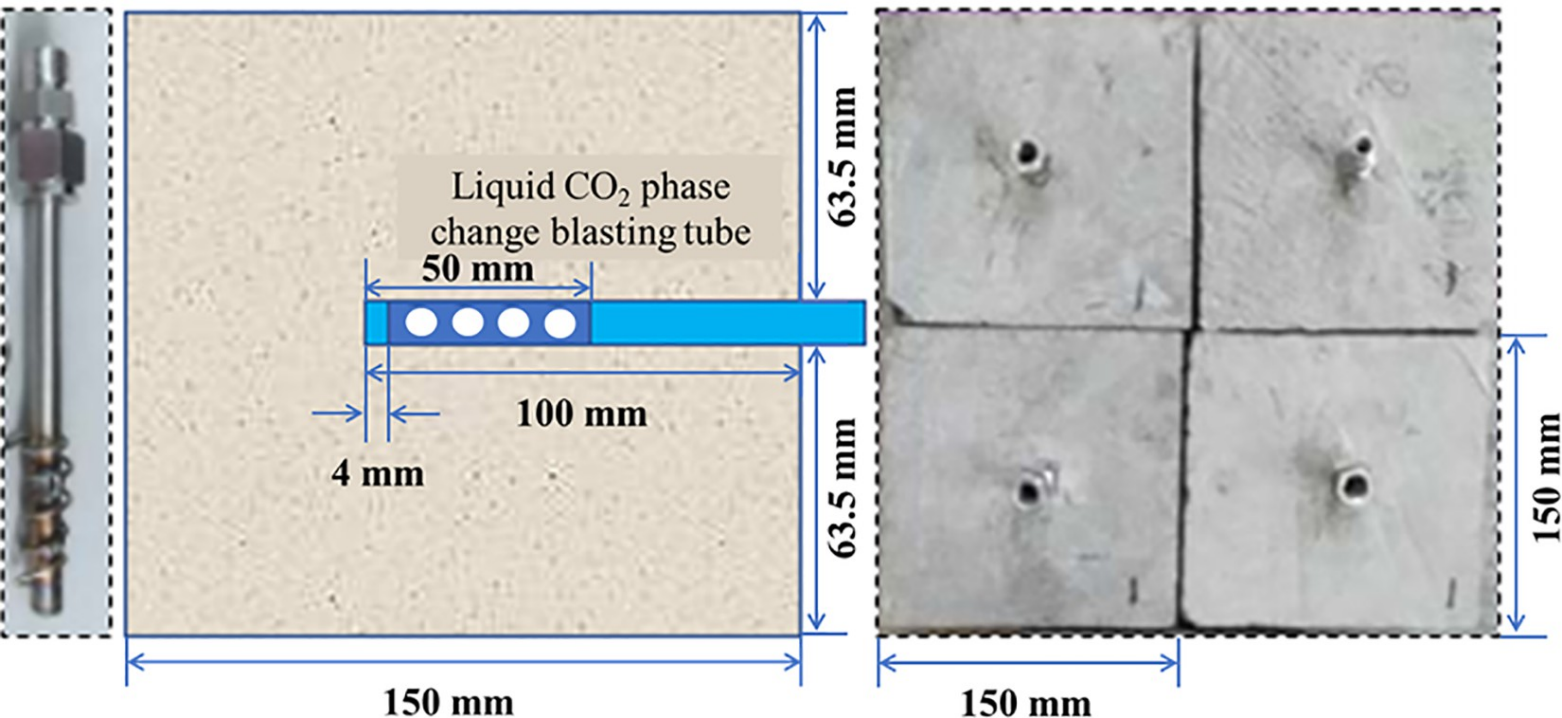
Preparation of model test specimen.

The production method of the coal sample model specimen is depicted in Fig 2. Sand, cement, and gypsum are stirred evenly according to the specified ratio. The mixture is then poured into a 150×150×150 mm^3^ cube mold, stirred, and shaken. Finally, the liquid CO_2_ release tube is installed in the mixed material to complete the production. The diameter of the blasting hole is 6.5 mm. Each hole is spaced 5 mm apart.

The size effect of the blast specimen is a critical factor in ensuring the reliability and representativeness of the experimental results. In the present study, the dimensions of the coal rock specimens were chosen based on a balance between experimental feasibility and the ability to simulate realistic conditions. The selected specimen size was designed to be large enough to minimize the influence of boundary effects while ensuring that the shock wave propagation could develop fully before reaching the specimen edges. This helps in replicating the stress wave distribution that would occur in an actual coal seam. To prevent the test from failing solely due to the impact of stress waves, the specimen size was carefully chosen to ensure that the energy dissipation process, including both the initial impact and the quasi-static expansion phase, could be adequately captured. If the specimen is too small, the stress waves generated by CO_2_ phase change may cause immediate failure, which would not reflect the intended fracture propagation mechanism.

### 2.3 Experimental device and experimental steps

**Fig 3:**
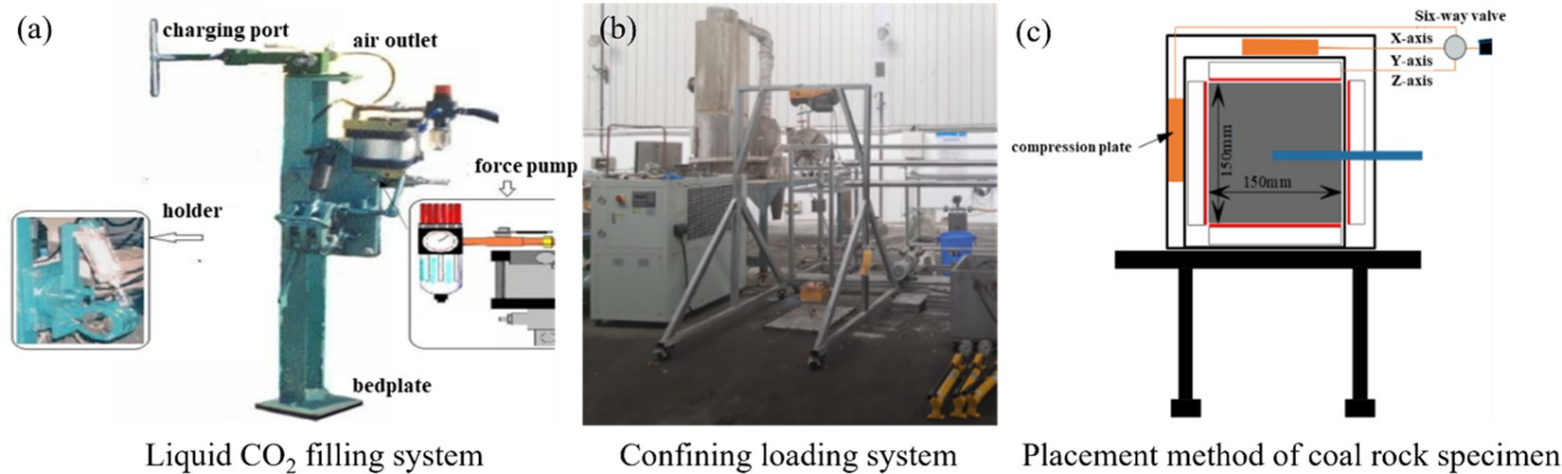
Experimental facility of liquid CO_2_ phases change fracturing. (a) liquid CO_2_ filling system; (b) confining loading system; (c) placement method of coal rock specimen.

The experiment was conducted on a small-scale liquid CO_2_ phase change blasting fracturing coal test bench, as depicted in Fig 3. The test bench primarily comprises a liquid CO_2_ phase change blasting system and a confining pressure loading system.

This experiment primarily investigates the deformation and crack morphology of coal-rock specimens from the blasting borehole to the specimen surface. To ensure the safety of operators during the experimental process, coal-rock specimens are sealed in a box throughout the entire experiment. However, this also results in the inability to use a high-speed camera to monitor the dynamic crack process of the coal-rock specimen surface in real time. The expansion of coal-rock surface cracks under confining pressure can only be determined by embedding strain gauges on the specimen’s surface. The expansion of coal-rock cracks near the fracturing hole is characterized by comparing crack expansion before and after fracturing.

Table 2 demonstrates the parameters of liquid CO_2_ phase change blasting experiments. The specific steps of the experiment are as follows:

1. As per Fig 3, strain gauges are arranged on the surface of the model specimen and placed in the three-axis loading chamber. The strain gauge is then connected with the data acquisition instrument and the high-pressure pipeline between the release tube and the pneumatic ball valve.
2. The triaxial stress loading of the sample is carried out by pushing the baffle using a manual booster pump.
3. The critical value for pressure release of the pneumatic ball valve is set, and the heating device is initiated to heat the liquid CO_2_ phase change generator. The experiment commences when the pressure reaches the critical pressure set by the pneumatic ball valve.
4. The data acquisition instrument is opened to detect the stability of the data channel, and the experiment starts when the pressure reaches the set critical value of the pneumatic valve.
5. After completing the experiment, the sample is removed, and its failure characteristics are analyzed. Once the phase change generator temperature naturally cools to room temperature, the experimental conditions are altered, and the above operation is repeated. In accordance with the actual conditions of Linhua Mine, this paper conducted an experiment on liquid CO_2_ phase change blasting fracturing coal under different triaxial stress conditions. The liquid CO_2_ phase change blasting pressure was set at 5.0, 7.5, and 10.0 MPa respectively.

**Table 2:** Parameters of liquid-CO_2_ phase change blasting experiments.

No.	blasting pressure (MPa)	ambient temperature (°C)	triaxial stress		
			σ_*x*_	σ_*y*_	σ_*z*_
Specimen 1	5.0	25	0	0	0
Specimen 2	5.0	25	6.2	4	3.6
Specimen 3	5.0	25	6.2	4	3.6
Specimen 4	7.5	25	6.2	4	3.6
Specimen 5	10.0	25	6.2	4	3.6

The selection of blasting pressure and triaxial stress conditions was based on multiple considerations to ensure that the experimental setup effectively represents realistic in-situ mining conditions and supports a systematic investigation of crack propagation behavior. The blasting pressures of 5.0 MPa, 7.5 MPa, and 10.0 MPa were chosen to represent a range of realistic in-situ conditions that are relevant for coal seam gas extraction. These pressures were sufficient to initiate and propagate fractures in coal rock while also allowing a comparison of fracture characteristics under varying blasting intensities. The triaxial stress was selected to simulate the geostress conditions encountered in deep underground coal seams. The chosen stress levels were designed to closely approximate the confining pressures experienced at different depths in coal mines, thereby allowing for a more realistic assessment of how confining stress affects fracture propagation.

Specimen 1 was tested without confining pressure, while Specimen 2 was subjected to a confining pressure. This comparison was designed to study the effect of confining pressure on crack initiation and propagation. Specimen 3, 4, and 5 were subjected to different blasting pressures of 5.0 MPa, 7.5 MPa, and 10.0 MPa, respectively, while keeping the confining pressure constant. This set of experiments aimed to assess the influence of increasing blasting pressure on fracture formation. By systematically varying either the confining pressure or blasting pressure, the study effectively isolates the impact of these parameters on crack behavior, providing clearer insights into the mechanisms of fracture propagation under different stress conditions.

## 3 Results and analysis

### 3.1 Failure mode of coal-rock model specimen

**Fig 4:**
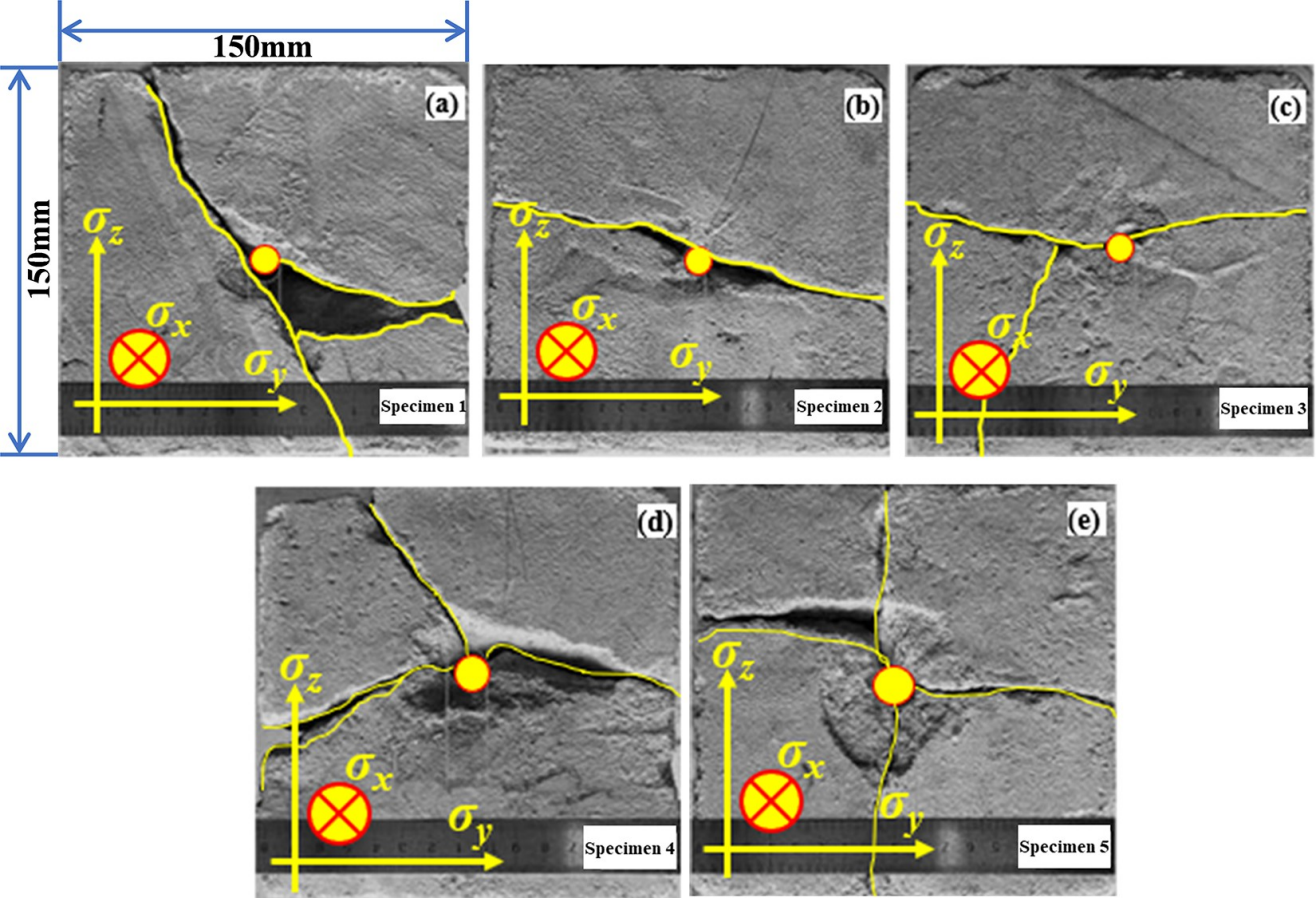
Failure pattern of the coal-rock model specimen surface after the fracturing experiments. (a) specimen 1; (b) specimen 2; (c) specimen 3; (d) specimen 4; (e) specimen 5.

Fig 4 shows the failure modes of liquid CO_2_ phase change blasting fracturing coal rock model specimens under different experimental parameters as shown in Table 2. The analysis reveals that the inner surface of each specimen within a certain range near the fracturing hole exhibits varying degrees of damage, which is attributed to the stress waves generated by the violent phase change blasting of liquid CO_2_ [73,74]. The vibration monitored in the test represents the dynamic response corresponding to the stress wave [75,76]. When comparing the failure modes of specimens 1, 2, and 3, it can be observed that the application of confining pressure reduces the expansion deformation rate of the specimen. In other words, the confining pressure significantly enhances the tensile strength and ductility of the coal-rock specimen, resulting in a significantly smaller crack zone in specimens 2 and 3 compared to specimen 1. Comparing the failure modes of specimens 3, 4, and 5 reveals that with an increase in liquid CO_2_ phase change blasting release pressure, the range of the crack zone around the borehole significantly increases, as does the number of cracks formed by strong impact. This indicates that the range of the phase change blasting crack zone is primarily determined by impact strength. A larger phase change blasting release pressure will produce a stronger shock wave, which will promote further expansion of the borehole.

### 3.2 Morphology and distribution characteristics of liquid CO_2_ phase change blasting

To study the crack morphology and distribution characteristics of coal rock after liquid CO_2_ phase change blasting, the failure morphology and crack distribution of the aforementioned five coal rock specimens were statistically analyzed and compared.

**Fig 5:**
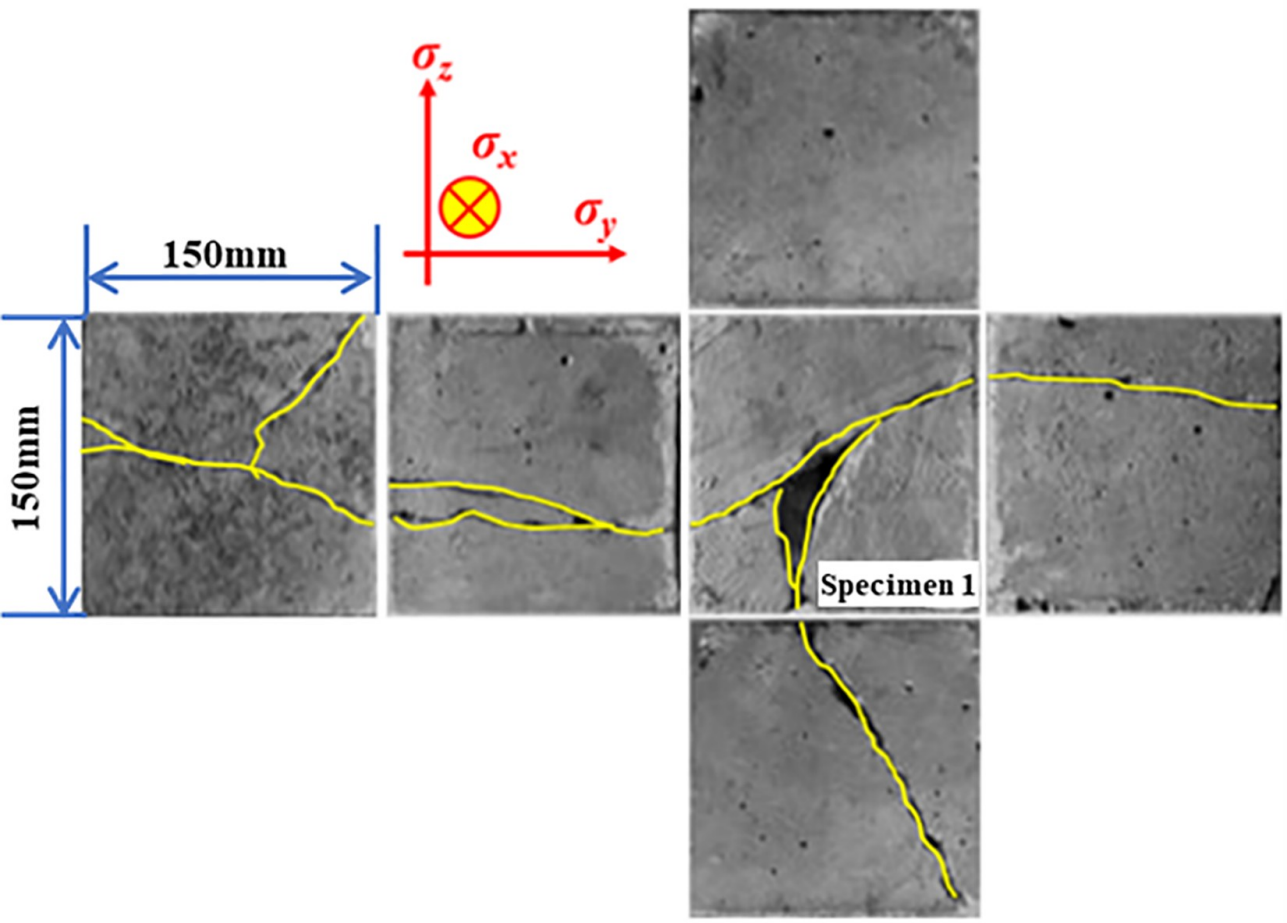
Cracks of each surface of specimen 1 after the fracturing experiment.

The crack morphology distribution of specimen 1 after fracturing failure is depicted in Fig 5. The analysis reveals that the specimen produces penetrating cracks in the axial direction of the borehole, and two surface cracks in the axial direction of the borehole result in bifurcation. The bifurcation of the axial surface through the crack leads to three surface cracks on four radial surfaces. This is primarily due to the heterogeneity of concrete, which causes new cracks around the borehole to expand along scattered cracks, thus causing the crack to turn.

**Fig 6:**
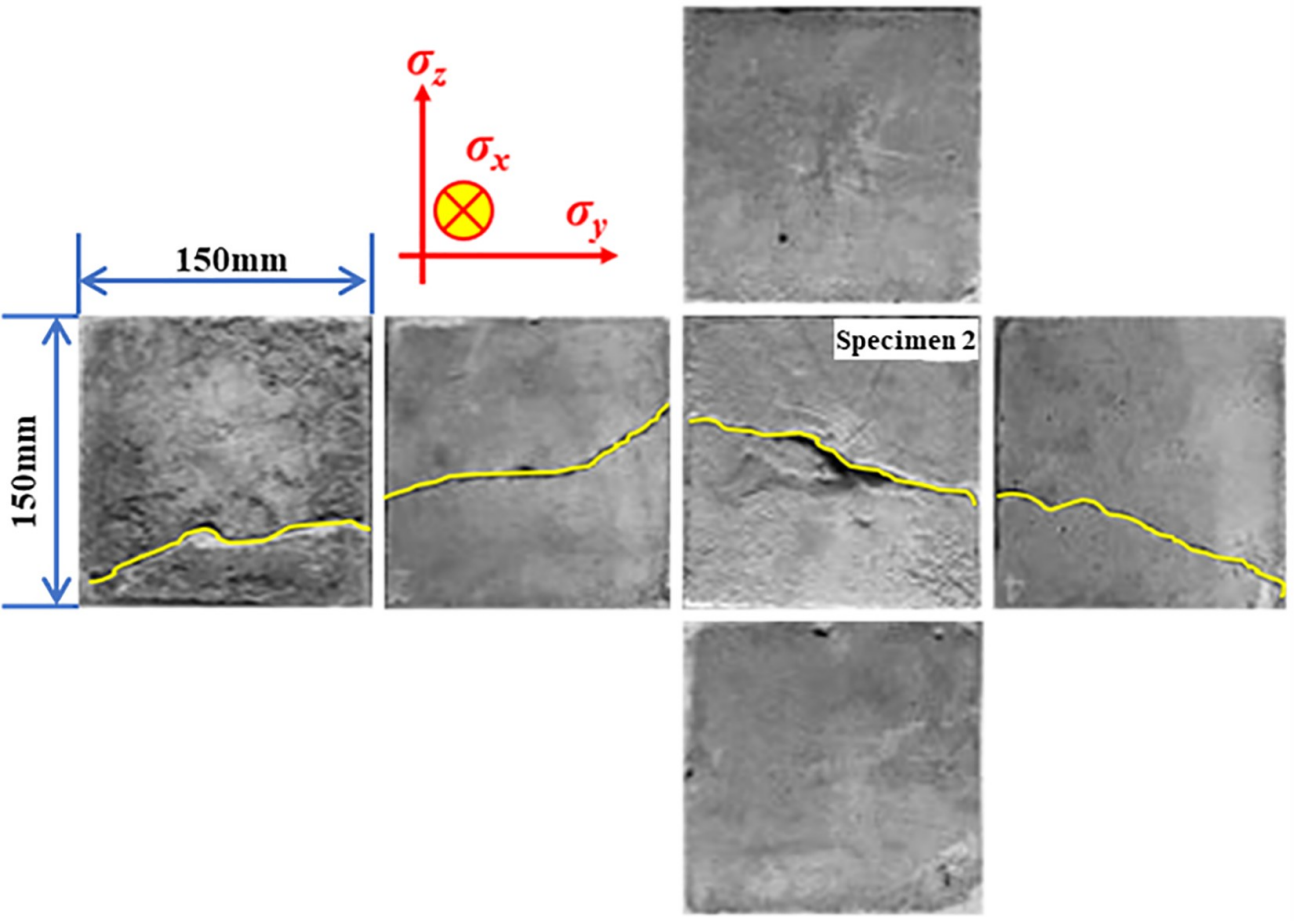
Cracks of each surface of specimen 2 after the fracturing experiment.

Fig 6 depicts the distribution of cracks on the surface of specimen 2 after the completion of the experiment. The analysis reveals that the distribution of cracks after fracturing of coal and rock specimens is essentially the same as that after hydraulic fracturing, based on the traditional maximum tensile stress criterion. In other words, the specimen produces main cracks along the direction perpendicular to the minimum principal stress. Compared with specimen 1, the number and direction of cracks produced by liquid CO_2_ phase transition crack change significantly after confining pressure is applied. This is primarily due to the increase in the minimum impedance of the crack propagation path caused by the application of confining pressure. Clearly, due to σy > σz, the crack propagation in the y-axis direction is limited, and the confining pressure results in a relatively flat crack surface on the specimen.

**Fig 7:**
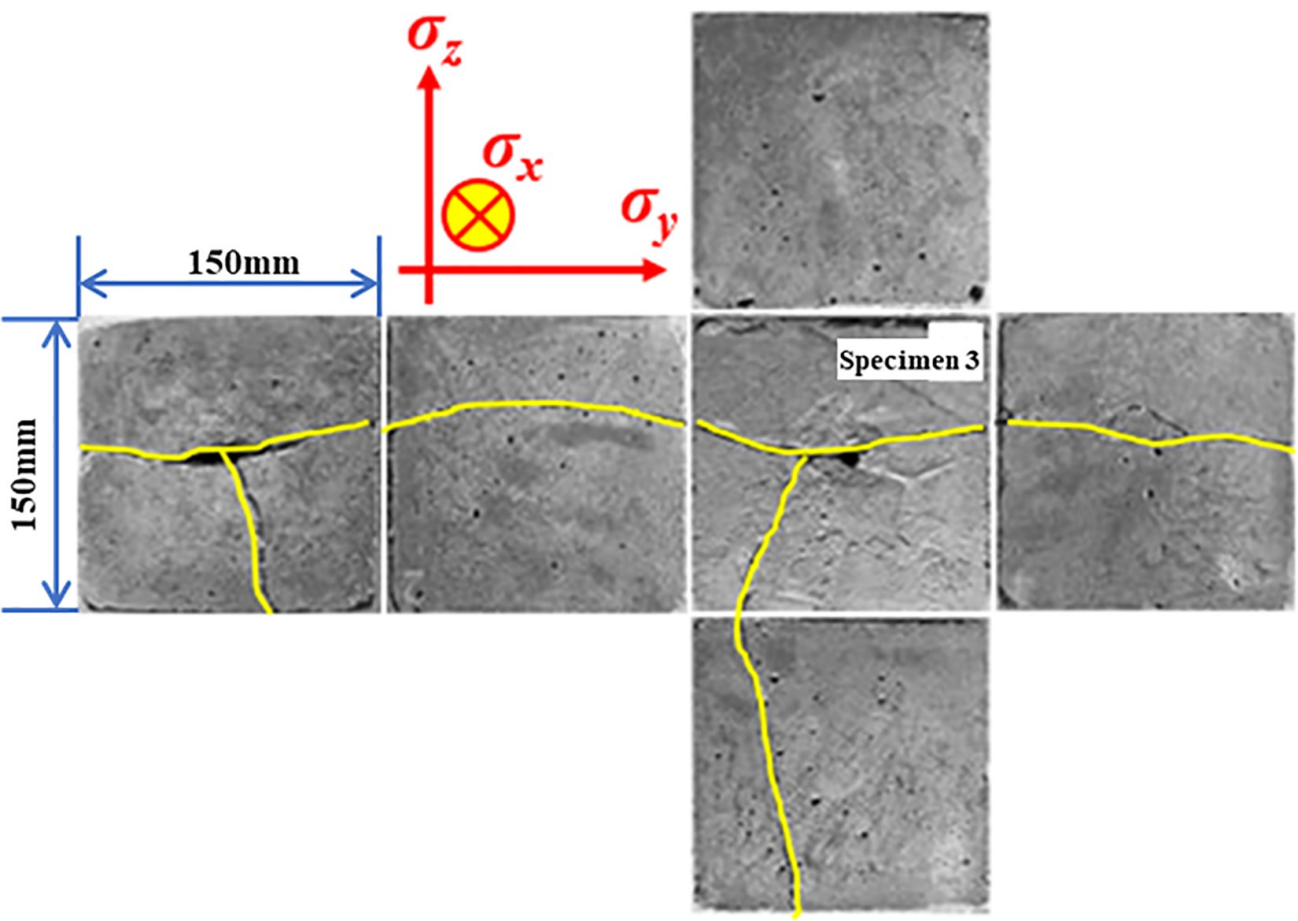
Cracks of each surface of specimen 3 after the fracturing experiment.

Fig 7 depicts the distribution of cracks on the surface of specimen 3 after the experiment. When comparing the crack distribution of specimen 1 and specimen 2, it can be observed that the expansion of cracks in specimen 3 still extends in the direction perpendicular to the minimum principal stress. However, due to the increase in liquid CO_2_ mass in specimen 3, the cracks on the axial surface of the borehole bifurcate. This is primarily due to the continuous filling of high-pressure gas phase CO_2_ into the cracks, leading to continuous expansion of the cracks and forming a new crack surface. This indicates that the phase change expansion of liquid CO_2_ also plays a crucial role in the phase change blasting process of liquid CO_2_.

**Fig 8:**
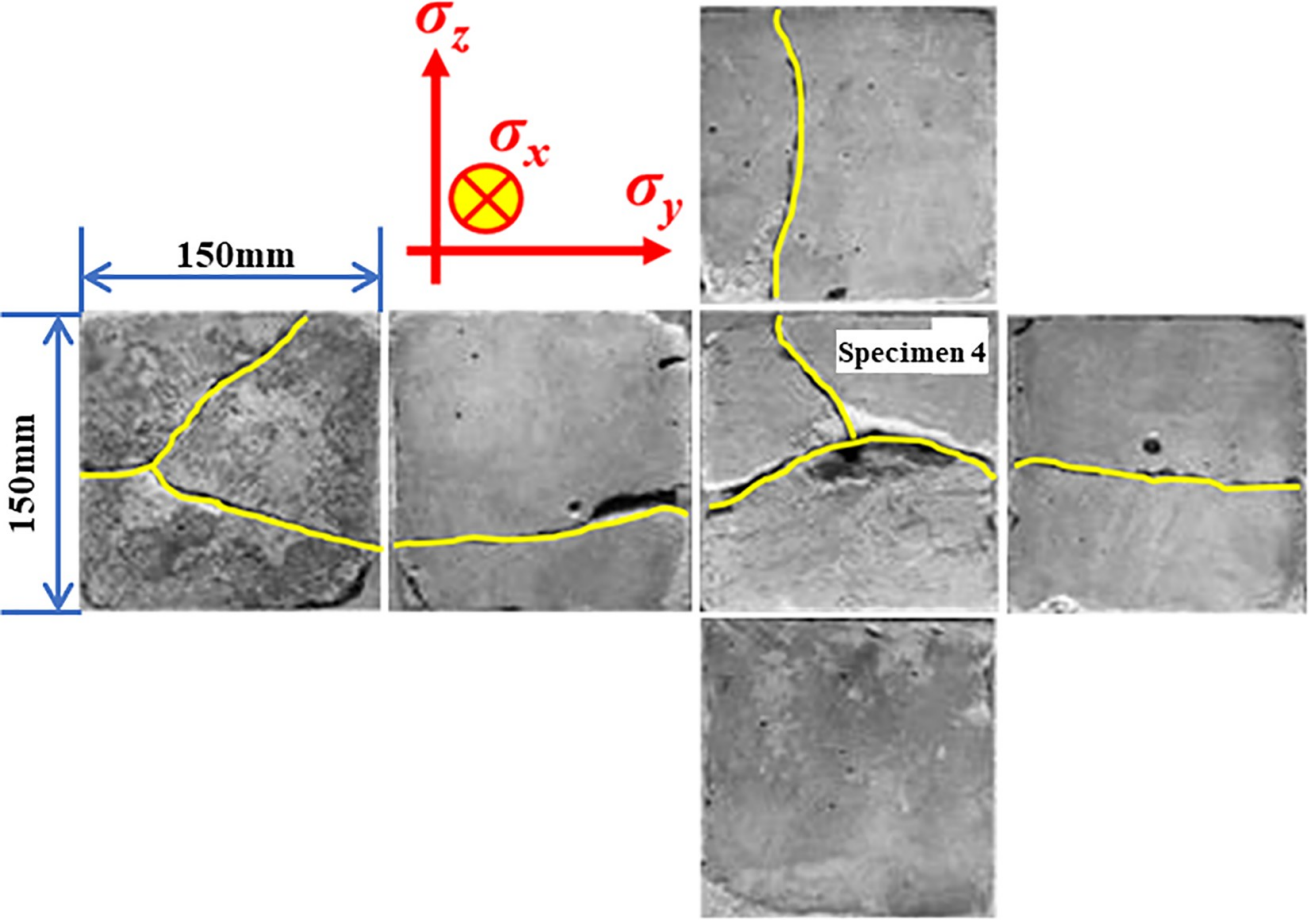
Cracks of each surface of specimen 4 after the fracturing experiment.

Fig 8 depicts the surface crack distribution of specimen 4 after the experiment. When compared with specimen 2 and specimen 3, it can be observed that with the increase in confining pressure, the angle between the crack caused by liquid CO_2_ phase change blasting and the direction of maximum principal stress increases, resulting in three main cracks appearing in the radial direction. The newly generated crack surface has gradually expanded to the surface of the specimen under the influence of gaseous liquid CO_2_, leading to the fracturing of the specimen. This occurs because the high-pressure liquid CO_2_ comes into contact with the newly generated crack surface of the specimen after phase transition, and the strong adsorption of CO_2_ causes it to enter and damage the pores. Additionally, the strong flow pressure also causes the new crack surface to continue weathering and corroding, further damaging the crack surface.

**Fig 9:**
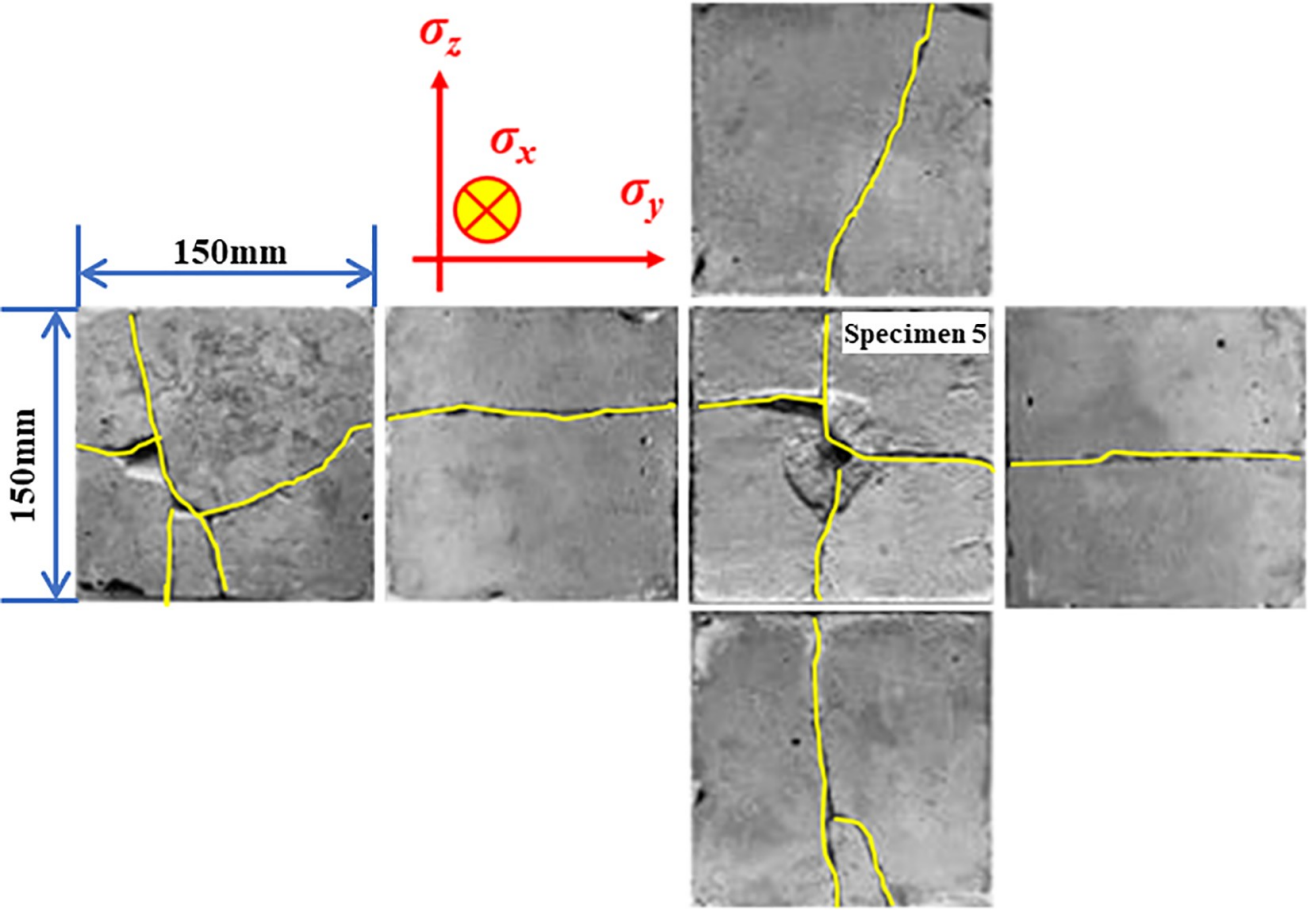
Cracks of each surface of specimen 5 after the fracturing experiment.

Fig 9 depicts the distribution of cracks on the surface of specimen 5 after the experiment. When comparing specimen 4 and specimen 3, it can be observed that with the increase in phase change pressure, the number of cracks on the radial surface of the borehole further increases, with 4 and 5 main cracks appearing on the two radial surfaces respectively [77]. The crack bifurcation appears in the axial plane of the borehole, meaning that the generation and development of the crack are no longer perpendicular to the direction of the minimum principal stress. This indicates that the number of cracks determined by liquid CO_2_ phase transition fracturing is no longer influenced by confining pressure.

The application of triaxial stress in specimens 2 and 3 led to more controlled and directional crack propagation, with cracks primarily forming perpendicular to the minimum principal stress. The presence of confining pressure limited crack expansion along specific axes, resulting in flatter and more uniform crack surfaces. This suggests that confining pressure plays a critical role in controlling crack propagation by enhancing directional constraints. Specifically, under confining pressure, cracks tend to propagate perpendicular to the minimum principal stress, which leads to a more predictable and less complex crack network. Further research is needed to quantify these effects under varying stress states and to understand their implications for optimizing blasting designs.

As blasting pressure increased from 5.0 MPa to 10.0 MPa, the number and complexity of cracks increased. Higher pressures caused cracks to form in multiple directions, including bifurcating along axial surfaces, and at the highest pressure, crack propagation was less influenced by the confining stress, leading to a more extensive and complex fracture network.

### 3.3 Cracks expansion during the process of liquid CO_2_ phase change blasting of coal and rock

In summary, the generation and expansion of cracks during the process of liquid CO_2_ phase transition fracturing of coal and rock appear to be influenced by the combined effects of two processes: shock wave and quasi-static stress, both generated by the phase transition of high-pressure liquid CO_2_ in the borehole [78]. While the experimental observations suggest a potential interplay between these processes, further research is needed to fully substantiate their individual contributions to crack propagation. The results from Sections 3.1 and 3.2 reveal that cracks form in distinct zones around the borehole, including the broken zone and crack zone, and these observations are supported by both analysis of crack patterns and distribution.

The inner surface near the borehole undergoes significant damage due to the high-energy shock wave generated during the phase change (as discussed in Section 3.1). Near the borehole, the initial principal stress in the two radial planes is in a compressed state, and the tangential compressive stress rapidly converts into tension. As a result, the area near the borehole is crushed to form a crushing area, which align with the observations in Section 3.1, where the confining pressure and release blasting pressure significantly influenced the extent of the crack zone and the number of cracks formed. After blasting, the specimen distributes numerous cracks of varying lengths along the weak surface and radial direction of the borehole. Outside the crushing zone, due to the gradual attenuation of the shock wave, further breakage of the specimen outside this zone is prevented.

In the crack zone, due to the strong tensile stress generated by the high-pressure CO_2_ wedge and given that the tensile strength of the coal rock specimen is significantly smaller than its compressive strength, crack expansion continues. Therefore, a crack zone may form where tangential tensile stress exceeds tensile strength. This process falls under a typical quasi-static mechanics category, with preferred cracks in the coal specimen extending along the direction of minimum impedance. Further away from the borehole, in the crack zone, the quasi-static stress plays a dominant role in the expansion of cracks. Section 3.2 demonstrated that the crack propagation in this zone occurs primarily in the direction perpendicular to the minimum principal stress. The analysis of the crack distribution in specimens 2, 3, and 4 showed that under the influence of confining pressure, the propagation direction and surface morphology of the cracks changed, corroborating the role of quasi-static tensile stress in extending the cracks. This is particularly evident in the bifurcation of cracks observed in the radial direction, as discussed in Sections 3.1 and 3.2.

Finally, areas outside the fault zone remain in a bidirectional compression state, halting further fault extension. Outside the crack zone, the elastic zone experiences limited crack growth due to the attenuation of shock wave energy. However, due to the limitation of specimen size, the boundary of the elastic zone could not be clearly defined. Nevertheless, the present experiment successfully identified the distribution of the complete broken zone as well as part of the crack zone.

In addition, previous studies have analyzed these zones by characterizing the energy distribution throughout the process [79]. Key results show that the broken zone is confined to a radius of approximately 0.045 m from the fracturing hole, while energy as the distance increases. The radial particle velocity was concentrated within a low-frequency range (0–100 Hz), and the CO_2_ gas pressure played a crucial role in the fracturing process, affecting the energy across different zones. These results corroborate well the crucial role of CO_2_ blasting pressure in affecting the crack propagation behavior across different fracture zones. Therefore, in the process of liquid CO_2_ phase change blasting, the energy distribution in the broken and crack zones is critical to understanding the crack propagation mechanism. The broken zone, located near the borehole, is characterized by intense localized damage due to the high-energy release of shock waves. In this region, the energy is rapidly dissipated through high-frequency vibrations, leading to significant fragmentation. In contrast, the crack zone exhibits a more complex energy distribution, primarily influenced by quasi-static stresses. The high-pressure CO_2_ creates a static wedging stress around the borehole, which drives crack propagation radially. Experimental observations indicate that energy is gradually released in the crack zone through stress concentration, resulting in crack growth predominantly along the direction of the minimum principal stress.

## 4 Discussion

Liquid CO_2_ phase change blasting fracturing enhanced permeability technology is a new type of enhanced permeability technology that injects liquid CO_2_ into coal rock under high pressure, inducing the initiation and expansion of internal cracks in coal rock layers, increasing the permeability of coal seams, and promoting the migration of CH_4_ and other gases in coal seams. The experimental results of this study demonstrate the formation of distinct zones: crushed zone and crack zone, due to the shock waves generated by the CO_2_ phase transition. With the increase of liquid CO_2_ phase change blasting release pressure, the crack range around the borehole increased significantly, and the number of cracks formed by strong impact also increased significantly. While these findings provide valuable insights into the crack propagation under liquid CO_2_ phase change blasting. These findings are a crucial step toward optimizing the technology for practical applications in coal seam gas extraction, increasing both efficiency and safety.

The experimental results demonstrate that increasing the liquid CO_2_ blasting pressure significantly enhances both the range and density of cracks formed around the borehole. In field applications, this suggests that higher blasting pressures can create a more extensive fracture network, which is beneficial for improving the permeability of low-permeability coal seams. However, careful consideration must be given to the structural integrity of surrounding coal to avoid excessive fracturing that may compromise stability. Moreover, the study highlights the critical role of confining pressure in controlling crack propagation. In field applications, confining pressure represents the in-situ stress conditions that vary with depth and geological characteristics. By applying confining pressure in the experiments, it was observed that the number of cracks and their expansion were significantly limited, which implies that in deeper coal seams with higher natural confining stresses, crack propagation may be more controlled and localized. This understanding is crucial for planning controlled blasting in deep mining environments to avoid undesired damage beyond the target area. Therefore, the findings suggest that optimizing blasting parameters, such as selecting appropriate CO_2_ pressures based on the target coal seam’s depth and stress state, can enhance the effectiveness of gas extraction while minimizing the risk of excessive damage. For instance, lower blasting pressures may be more suitable for shallow seams with low confining pressures to avoid over-fracturing, whereas higher pressures can be used in deep seams with higher confining stresses to achieve effective permeability enhancement.

Although field experiments were not conducted in this study, the laboratory results offer a scientific basis for future field testing and development. Once validated through on-site experiments, the insights gained here could guide the effective arrangement of boreholes and the strategic application of liquid CO_2_ phase change blasting fracturing to maximize gas extraction and minimize the risk of gas-related incidents. Therefore, the delineation of broken and crack zones as well as the morphology of cracks show high similarity with the actual field conditions in this experiment, indicating that the experimental results have strong reference value. This similarity provides a basis for further verifying the crack extension law under field conditions, and also lays a foundation for subsequent research and optimization of experimental design. In the future research, the extension law of broken and crack zones under different conditions can be further explored by modifying the experimental parameters, so as to better guide the practical engineering.

While this study provides valuable insights into the crack propagation behavior under different blasting pressures during liquid CO_2_ phase change blasting, there are certain limitations. First, the experiments were conducted in a laboratory environment with controlled parameters, which may not fully replicate the complex conditions found in actual coal seams. The lack of field experiments limits the direct applicability of the results to real-world operations. Furthermore, the study primarily focused on the effects of varying CO_2_ blasting pressures without extensively exploring other influential factors, such as varying temperature or coal seam gas pressures. Therefore, to further validate the findings and extend their practical applicability, future research should incorporate field experiments in actual coal mines to assess the effectiveness of CO_2_ phase change blasting fracturing under real-world conditions. Additionally, exploring the combined effects of other variables, such as temperature, on crack propagation will provide a more comprehensive understanding of how CO_2_ phase change blasting fracturing technology can be optimized for various geological settings. Furthermore, while the current study primarily focused on qualitative analysis of the blasting cracks, future research should include more quantitative analyses, such as detailed measurements of crack length, width, density, and volume. Incorporating advanced monitoring techniques, such as three-dimensional imaging and real-time crack propagation monitoring, will help obtain more precise quantitative data. In the end, to improve the accuracy and relevance of future studies, it is suggested that a more comprehensive approach be taken by incorporating advanced monitoring techniques, such as real-time crack propagation monitoring and three-dimensional fracture modeling.

## 5 Conclusions

Through the experiment conducted on a small liquid CO_2_ phase change blasting coal test device, the characteristics of crack propagation of liquid CO_2_ phase change blasting fracturing coal with different blasting pressure were elucidated. The main conclusions are as follows:

1. Under the same confining pressure and volume, with the increase of liquid CO_2_ phase change blasting release pressure, the crack range around the borehole increased significantly, and the number of cracks formed by strong impact also increased significantly. This suggested that a higher phase-change release pressure produced a stronger shock wave, which promoted further borehole enlargement.
2. By analyzing the surface cracks of the specimens, it is found that when the CO_2_ phase change blasting pressure is constant, the number of cracks generated under confining pressure is significantly less than that without confining pressure. The crack propagation gradually becomes perpendicular to the direction of the minimum principal stress. Under constant confining pressure conditions, the number of initial cracks is not affected by volume, and crack bifurcation increases. The number of cracks on the radial surfaces of the borehole increased further with increasing CO_2_ phase change blasting pressure, with four and five major cracks appearing on the two radial surfaces, respectively.
3. The process of crack generation and expansion during liquid CO_2_ phase change blasting in coal and rock appears to be influenced by the combined effects of shock waves and quasi-static stress resulting from the high-pressure CO_2_ phase transition in the borehole. Cracks form in distinct zones: the broken zone, where shock waves cause severe crushing near the borehole; the crack zone, where quasi-static tensile stress drives crack propagation. Higher confining and CO_2_ blasting pressures increase crack propagation.
4. The study demonstrates that liquid CO_2_ phase change blasting significantly influences crack propagation characteristics in coal and rock under varying pressure and stress conditions. Increasing the blasting pressure led to a significant increase in both crack range and number, indicating that higher blasting pressures can effectively enhance coal seam permeability. These findings provide a scientific basis for optimizing CO_2_ blasting design, with important practical implications for improving coal seam gas extraction efficiency and ensuring rock stability.
